# “Blue Sky Effect”: Contextual Influences on Pupil Size During Naturalistic Visual Search

**DOI:** 10.3389/fpsyg.2021.748539

**Published:** 2021-12-21

**Authors:** Steven M. Thurman, Russell A. Cohen Hoffing, Anna Madison, Anthony J. Ries, Stephen M. Gordon, Jonathan Touryan

**Affiliations:** ^1^US DEVCOM Army Research Laboratory, Human Research and Engineering Directorate, US Army Research Laboratory, Aberdeen Proving Ground, MD, United States; ^2^DCS Corporation (United States), Alexandria, VA, United States

**Keywords:** pupil size, luminance, pupillary light response, pupillometry, active visual search, virtual environment

## Abstract

Pupil size is influenced by cognitive and non-cognitive factors. One of the strongest modulators of pupil size is scene luminance, which complicates studies of cognitive pupillometry in environments with complex patterns of visual stimulation. To help understand how dynamic visual scene statistics influence pupil size during an active visual search task in a visually rich 3D virtual environment (VE), we analyzed the correlation between pupil size and intensity changes of image pixels in the red, green, and blue (RGB) channels within a large window (~14 degrees) surrounding the gaze position over time. Overall, blue and green channels had a stronger influence on pupil size than the red channel. The correlation maps were not consistent with the hypothesis of a foveal bias for luminance, instead revealing a significant contextual effect, whereby pixels above the gaze point in the green/blue channels had a disproportionate impact on pupil size. We hypothesized this differential sensitivity of pupil responsiveness to blue light from above as a “blue sky effect,” and confirmed this finding with a follow-on experiment with a controlled laboratory task. Pupillary constrictions were significantly stronger when blue was presented above fixation (paired with luminance-matched gray on bottom) compared to below fixation. This effect was specific for the blue color channel and this stimulus orientation. These results highlight the differential sensitivity of pupillary responses to scene statistics in studies or applications that involve complex visual environments and suggest blue light as a predominant factor influencing pupil size.

## Introduction

In classic cognitive pupillometry studies, it has been critical to equate luminance across stimuli and/or experimental conditions to isolate cognitive influences on pupil size and ensure that results are not driven by confounds due to variable luminance. While this careful control of luminance has led to a great deal of knowledge about the relationship between pupil size and cognition (Aston-Jones and Cohen, 2005; Sirois and Brisson, 2014; Mathôt, 2018; Hoffing et al., 2020; Joshi and Gold, 2020), it also limits the ability to generalize research findings to contexts in which luminance cannot be controlled. For example, it would be unfeasible to control for luminance in real-world tasks and would impede the study of naturalistic gaze behavior in experiments using complex stimuli, to replicate the spatial variation in local contrast and luminance that occurs in the natural world (Frazor and Geisler, 2006).

The pupillary light response (PLR) represents a predictable, ballistic change in pupil size whenever there is a sudden change in luminance (Korn and Bach, 2016). As a rule, the pupil constricts and reduces in size whenever there is a sufficient increase in brightness and it dilates whenever there is a sufficient decrease in brightness, albeit more slowly than constrictions. The PLR is also a characteristically sluggish response that reaches its peak between 500 and 1,000 ms after a change in luminance (Mathôt, 2018), and only gradually returns to pre-stimulus baseline after several seconds. Gaps remain, however, in the understanding of how the pupil responds to light because it is still unclear how output from rods, cones, and intrinsically photosensitive retinal ganglion cells (ipRGC) are integrated to drive pupil size changes. For example, evidence from individuals with nonfunctioning rod and cone photoreceptors (Czeisler et al., 1995; Lockley et al., 1997) and transgenic mice without photosensitive RGCs (Lucas et al., 2001, 2003) suggest that both types of retinal cells contribute to the PLR; however, much less is understood about their relative contributions to the PLR, especially in uncontrolled settings with naturalistic and dynamic visual scenes.

It is well known that each type of photoreceptor has a different spectral sensitivity (Stockman et al., 1993; Do and Yau, 2010; Neitz and Neitz, 2011) and that these light-sensitive cells are non-uniformly distributed across the retina (Curcio et al., 1991; McDougal and Gamlin, 2010; Lee et al., 2017). Recent work leveraging the silent substitution method, which can selectively modulate the excitation of ipRGCs, rods, and the three cones separately (or combined), suggests that color signals influence the pupil response differently (Barrionuevo et al., 2014; Barrionuevo and Cao, 2016). For example, Bonmati-Carrion et al. (2018) found that monochromatic and combined monochromatic light had a differential influence on the strength of the PLR depending on the wavelength. Specifically, blue light (479 nm) resulted in a significantly faster velocity of constriction than purple (437 nm) or red (627 nm) light. Further complicating the matter, the PLR can also be modulated by contextual information, such as the expectation of a luminance increase, where participants showed increased PLRs to brightness illusions, such as the Helmholtz-Kohlrausch effect (Laeng and Endestad, 2012; Wood, 2012; Zavagno et al., 2017; Suzuki et al., 2019) and pictures of the sun (Naber and Nakayama, 2013; Castellotti et al., 2020), despite stimuli being equiluminant. By furthering our understanding of how dynamic visual scene statistics, such as luminance, spectral content (i.e., color), and context influence pupil size, it may be possible to better account for their contribution to the pupillary signal and improve estimation of residual cognitive-based effects on pupil size.

The present study aims to further our understanding of pupillary dynamics in a visually rich environment that involves an unconstrained navigation and visual search task in a 3D virtual environment (VE). Specifically, we focus on understanding how visual patterns modulate pupil size, where luminance changes dynamically over time even while behaviors and cognitive processes may also be concomitantly influencing pupil size. We investigated the influence of the spatial location of luminance in relation to the fovea as well as the spectral wavelength on pupil size changes. We hypothesized that the influence of luminance on pupil size would be greatest for pixels near the fovea and would reduce with eccentricity in a radial manner. This hypothesis is consistent with previous work indicating that the strength of the PLR is reduced as a function of eccentricity (Crawford and Parsons, 1936; Legras et al., 2018; Hu et al., 2020), which may be attributed to the diminishing distribution of photoreceptors farther away from the fovea. We also hypothesized that the relationship between luminance and pupil size would vary by wavelength, consistent with prior work indicating that blue colored light is perceived as being brighter (Suzuki et al., 2019) and can have a distinct influence on the PLR (Bonmati-Carrion et al., 2016). To investigate both hypotheses, we examined correlations between pupil size and intensities in the red, green, and blue (RGB) color channels derived from the sequence of images seen on the screen throughout the task. We computed pixel-wise correlation maps to visualize the correlation between pupil size and every pixel in a broad window (approximately 14 degrees visual angle) surrounding gaze position.

Foreshadowing our results from Experiment 1, the correlation maps surprisingly revealed that pixels closest to fixation actually varied the least with pupil size, contrary to our hypothesis of a foveal bias. The maps instead uncovered a distinct spatial pattern to indicate a significant contextual effect in which blue pixels, specifically located above the gaze position, had a disproportionate influence on pupil size. These results were interpreted to be related to a blue light from above or “blue sky effect,” reasoning that from an ecological perspective it would make sense for the brain to anticipate a brightness change whenever there is a visual pattern resembling a blue sky overhead due to its association with daytime and sunlight (Laeng and Endestad, 2012; Naber and Nakayama, 2013; Castellotti et al., 2020). In Experiment 2, we performed a controlled laboratory experiment that paired a gray patch with luminance-matched red, green, and blue patches located either on the top, bottom, left, or right relative to the control (gray). Results of this follow-on study confirmed our hypothesis, demonstrating a significant and highly specific “blue sky effect” on the PLR.

### Participants

Thirty-eight subjects were recruited from the Los Angeles area to participate in this study (Enders et al., 2021). Four subjects were missing a majority of eye-tracking data due to miscalibration or some technical error and were not included in this analysis, leaving a final sample of 34 subjects for this report (12 females, 22 males, age range = 19–64 years, mean = 39.5 ± 14.6 years). All subjects were at least 18 years of age or older and able to speak, read, and write English. All subjects signed an Institutional Review Board approved informed consent form prior to participation (ARL 19–122) and completed a web-based pre-screen questionnaire containing eligibility, demographic, and game-use questions. All subjects had normal hearing and reported normal or corrected-to-normal visual acuity and color vision. Additional visual acuity screening was conducted in-lab to ensure better than 20/40 vision using a standard Snellen Chart. Subjects were asked to read the 20/40 line of the Snellen chart and were allowed to participate if they made one mistake or less (the clinical Snellen test allows patients to make up to two mistakes on a line to be classified as that level of visual acuity). Normal color vision was assessed with a 14-plate Ishihara color test. Any subject who did not pass the entire screening process was not included in the study.

### Task and Procedure

Subjects completed demographic and survey questionnaires while being fit with an EEG cap prior to entering a whisper room (WhisperRoom Inc. MDL 4284 E) to undergo eye-tracking calibration and completed additional questionnaires pre- and post-tasks. The whisper room is a sound- and light-controlled chamber, where the only ambient lighting was provided by the computer screen. During the experimental session, subjects participated in four separate tasks including (i) classic rapid serial visual presentation (RSVP) target detection task (20 min), (ii) free viewing task to familiarize subjects with navigating the virtual environment (up to 12 min), (iii) the main free viewing visual search and navigation task (up to 20 min), and (iv) memory recall task (up to 15 min). All tasks were run with custom software using Unity 3D (Unity Technologies). Further documentation of the task and experimental design is described in previously published work (Enders et al., 2021). However, only results from pupillometry during the visual search and navigation task (iii) are described here.

**Figure 1:**
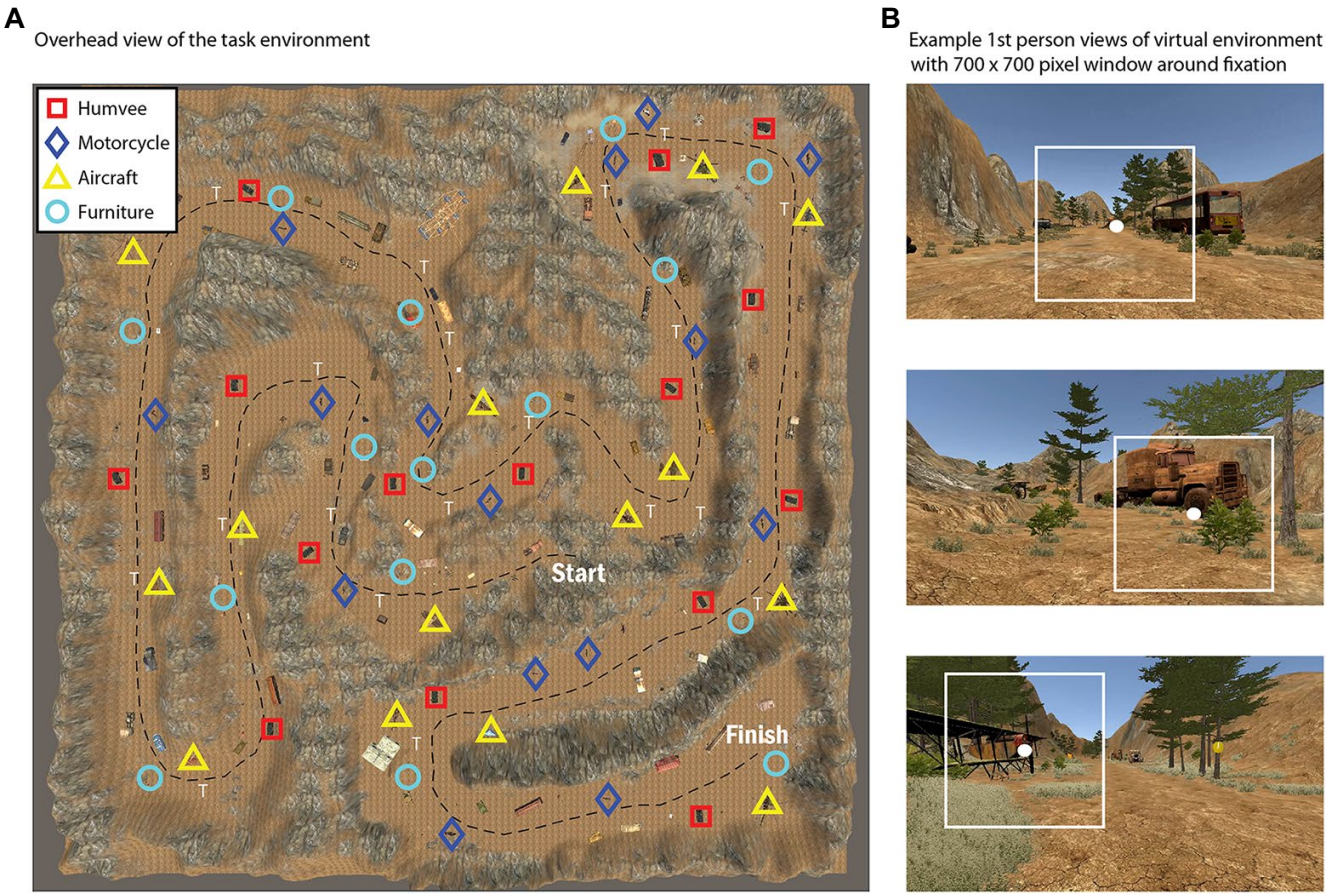
Overhead view of the task environment during the active visual search and navigation task **(A)**, with symbols representing the location of various target types (Humvee, Motorcycle, Aircraft, or Furniture). For further illustration, **(B)** shows selected snapshots of the first person view during the task with a 700 × 700 pixel box surrounding fixation.

In the visual search and navigation task, subjects were asked to freely navigate a virtual environment with the goal of searching for, and mentally counting, target objects from one of four categories that was randomly assigned to them (i.e., Aircraft, Motorcycle, Humvee, or Furniture). Subjects started at the same position in the virtual environment and all of the possible targets (15 total for each condition) were evenly distributed throughout the environment by experimenters to control the density of objects in each area (Figure 1A). Subjects had up to 20 min to identify and mentally keep count of the number of targets encountered using w/a/s/d keys for movement through the environment, and the mouse to control camera orientation to change heading direction and simulate head rotations. The task was performed on a computer monitor with a resolution of 1,920 × 1,080 pixels. Subjects were seated in a chair without a head or chin restraint and were asked to limit chair and body movements throughout the task. We used distance estimates from the eye-tracking system to confirm that subjects complied with this instruction. We found that subjects were positioned on average 62.1 cm from the screen (range = 55.0–78.0 cm) with a mean SD of 1.3 cm (range = 0.3–3.3 cm) over time.

About 8 min into the session an auditory Math Task was administered in which subjects were instructed to remember and report the sum of the numbers. Data collected during the math task, corresponding to 2.0 s (+0.47 s) on average, was cut from the time series data and omitted from analysis in order to reduce confounds associated with cognitive load and multi-tasking. Therefore, the main task during which we analyzed data in this report consisted of only the active visual search task, which had a mean duration of 10.4 min (+2.53 min). In this report, we leveraged this unique data set and the capability of replaying the entire set of visual scenes experienced by each subject to examine the relationship between pupil size and dynamic scene statistics irrespective of task-related cognition and behavior.

### Luminance Measurements

Screen luminance measurements were obtained with a SpectraScan Spectroradiometer PR-745. We wrote a program in Matlab (2019a, The MathWorks, Natick, MA) using the Psychophysics Toolbox 3.0.16 (Brainard, 1997; Pelli, 1997) to step through each of the 8-bit RGB color channels from 0 to 255 in increments of 3, displaying the color on the full screen until the spectroradiometer collected luminance measurements in units of cd/m^2^. When recording with the spectroradiometer, we matched the experimental setup to when subjects performed the task, and the distance of the device to the computer screen was positioned to match the average subject eye height and distance (62 cm) from the monitor. We fit the screen luminance data with an exponential function to estimate the best gamma parameter for transforming RGB color space into luminance space. Each color channel was best-fit by a slightly different gamma value (red gamma = 2.24, green gamma = 2.23, and blue gamma = 2.22). Prior to the experiment, we did not gamma correct the monitor by linearizing the color lookup table; instead, we applied this transformation *post hoc* to pixel intensities in each color channel, converting the images from 8-bit (0–255) color space to luminance space as a first step prior to subsequent analyses. We will use the term RGB luminance to reference this transformed image data.

### Eye-Tracking Data Collection

Binocular eye-tracking data (300 Hz) were collected with a Tobii Pro Spectrum mounted on the bottom of the computer monitor. Prior to the main task, we used a standard five-point calibration procedure to ensure proper calibration of the eye-tracking system. The Tobii Pro Spectrum recorded binocular estimates of pupil size and gaze position, while eye-tracking data were synchronized with game state (i.e., positions of players and objects in the Unity environment), keyboard, and mouse data using the Lab Streaming Layer (LSL) protocol (Delorme et al., 2011; Preuschoff et al., 2011; Kothe and Makeig, 2013; Kothe, 2014). The Tobii Pro Spectrum is reported to have an average binocular accuracy of 0.3° and binocular precision (root mean square) of 0.07° (Tobii Pro, 2018).

### Data Preprocessing

*Post hoc* data analysis involved generating the sequence of full-screen “snapshots” to replay the sequence of visual stimulation on the screen as subjects freely explored the virtual environment (Figure 1B). We generated the snapshot associated with every 10th frame for an effective temporal resolution of 12 Hz (screen refresh rate was 120 Hz), exporting the images in .png format. To save disk space, the sequence of images was then compressed in Matlab using the built-in MPEG video encoder, reducing the file size footprint by about 100x from ~100 gb for all .png images to a video of ~1 gb per subject. To ensure that video encoding did not introduce significant artifacts to the image data, we compared RGB pixel values of the original .png images to the compressed video frames and found that over 99% of pixel intensity differences were within a range of +12, a criterion that corresponds to 5% of the entire 8-bit color spectrum (0–255).

We used gaze position data output from the Tobii Pro Spectrum to extract local image statistics in relation to gaze position over time. Signal loss due to blinks and dropouts in the gaze position data corresponded to 13.9% (STD = 11%) of the data overall. The range of missing data due to signal loss across subjects was 2.4–41.1%. To investigate whether this data loss influenced the reported pattern of results, we analyzed data from the subset of 29 subjects that had less than 25% data loss (omitting the five subjects with greater than 25% data loss) and the overall pattern and statistical significance of group results were not affected whether including or excluding these subjects. Thus, these subjects were included in the presented results.

Instead of averaging pupil size data from the two eyes, which can introduce artifacts (e.g., abrupt discontinuities) when data are missing from one eye but not the other (due to baseline differences between the two eyes), we selected *a priori* to analyze the eye with the least amount of missing data (e.g., due to blinks and signal dropout due to eye/head rotations). Estimates of pupil size from commercial eye trackers can be noisy due to challenges in fitting the pupil region with an ellipse in the presence of eye lashes, partial eye closures, squinting, eye rotations, and other factors. These factors can sometimes introduce artifacts that appear as very abrupt and large changes in pupil size from one sample to another that are physiologically unrealistic. To reduce these artifacts in the pupil time series, we used an iterative velocity-based approach that examined the overall distribution of velocities over time and first identified all values that were greater than +2 SDs away from the mean and replaced them with not a number (NaN). It then used a more stringent criterion on the second iteration to remove values greater than +2.5 SDs from the mean to remove any remaining large outliers. These missing data points were then filled-in using linear interpolation of nearby data points.

A blink causes the eyelid to momentarily occlude the eye and causes a brief signal dropout because the eye is no longer visible to the tracker. Blinks were defined by short sequences of signal dropout that ranged from 50 to 500 ms of contiguously missing data. Missing data due to blinking were linearly interpolated using the best practice of also removing several data points (up to 50 ms) pre-blink and post-blink to remove potential artifacts due to partial eye closure surrounding each blink. We used the procedure published in the PRET toolbox (Denison et al., 2020), which is based on the technique published by Mathôt (2013). This technique first smooths the data with an 11 ms Hanning window and uses a velocity-based threshold to detect the onset and offset of each blink within a time window of +50 ms surrounding the epoch of missing data. The interpolated pupil size data and raw gaze position data were then downsampled to the same temporal resolution as the snapshot images (12 Hz) for subsequent analyses.

### Data Analysis

We computed correlation maps representing the Pearson correlation coefficient between pupil size and every pixel within a large region (700 × 700 pixels) centered on gaze position (+350 pixels, or approximately 7.6 deg. in each direction) derived from the snapshot images. To account for the expected time delay between changes in brightness/darkness and changes in pupil size due to the well documented sluggishness of the PLR (Mathôt, 2018; Denison et al., 2020), we used an empirical approach to estimate an appropriate time delay by cross-correlating pupil size with the RGB time series, and examining the peak temporal offset that maximized the correlation. We repeated this for every color channel (three channels) and subject (34 subjects), which resulted in a normal distribution of time lags with a mean = 469.4 + 140 ms and median = 500 ms (i.e., six frames at 12 Hz). This value is within the range of expectations according to prior literature (Ellis, 1981). Based on these results, we decided to fix the temporal offset at 500 ms for all subsequent analyses prior to computing correlation coefficients by shifting the pupil time series forward by six frames (500 ms) relative to RGB luminance. Of note, we performed follow-up analyses to investigate the influence of temporal offsets by varying the length of the temporal offset between 0 (no pupil lag) and 1,000 ms and found that the overall pattern of results was highly robust to the choice of lag value.

For computational tractability with these rather large image stacks, we downsampled the images by a factor of 10, from 70 0× 700 to 70 × 70 pixels, where each pixel in the correlation map was associated with the average intensity of a 10 × 10 block of pixels (approximately 0.2 deg.) with reference to the original resolution. When the gaze position was too close to the edge of the screen (i.e., within 350 pixels), we replaced pixels in the square window that would have fallen off-screen as NaNs in the image stack so they would not be incorporated into the correlation analysis. The total amount of missing image data (NaN values) due to fixations near the edge of the screen was 6.47%, and the percentage reached a maximum of 18% for pixels at the very top of the images. A figure representing the proportion of NaN values across space resulting from this procedure is shown in Supplementary File 1.

Because correlation coefficients are distributed non-normally, we performed a Fisher Z transform prior to computing group-level statistics (Dunn and Clark, 1969; Lenhard and Lenhard, 2014). We examined the consistency of spatial patterns in the correlation maps at the group level by averaging Fisher Z-transformed correlation maps across subjects and performing one-sample *t*-tests on a pixel-by-pixel basis comparing the distribution of values to the null hypothesis of 0 (no correlation). Due to the large number of pixels (4,900) included in this analysis, we used the procedure from Benjamini and Hochberg (1995) commonly used in fMRI to control the false discovery rate (FDR). We set a conservative criterion of FDR = 0.01 for analysis of pixels in the correlation maps.

To compare between each pair of color channels directly, we computed dependent-samples correlations (Williams, 1959; Steiger, 1980), as recommended by Steiger (1980), which tested the hypothesis that a pair of color channels was equally correlated with pupil size using a t-distribution. This statistical test is appropriate in such cases when two repeated-measures variables (i.e., two color channels) derived from the same individual are correlated with a third variable (i.e., pupil size). High *t*-values provide evidence to reject the null hypothesis and indicate a significant difference in the strength of the correlation between pupil size and one color channel vs. another color channel.

**Figure 2:**
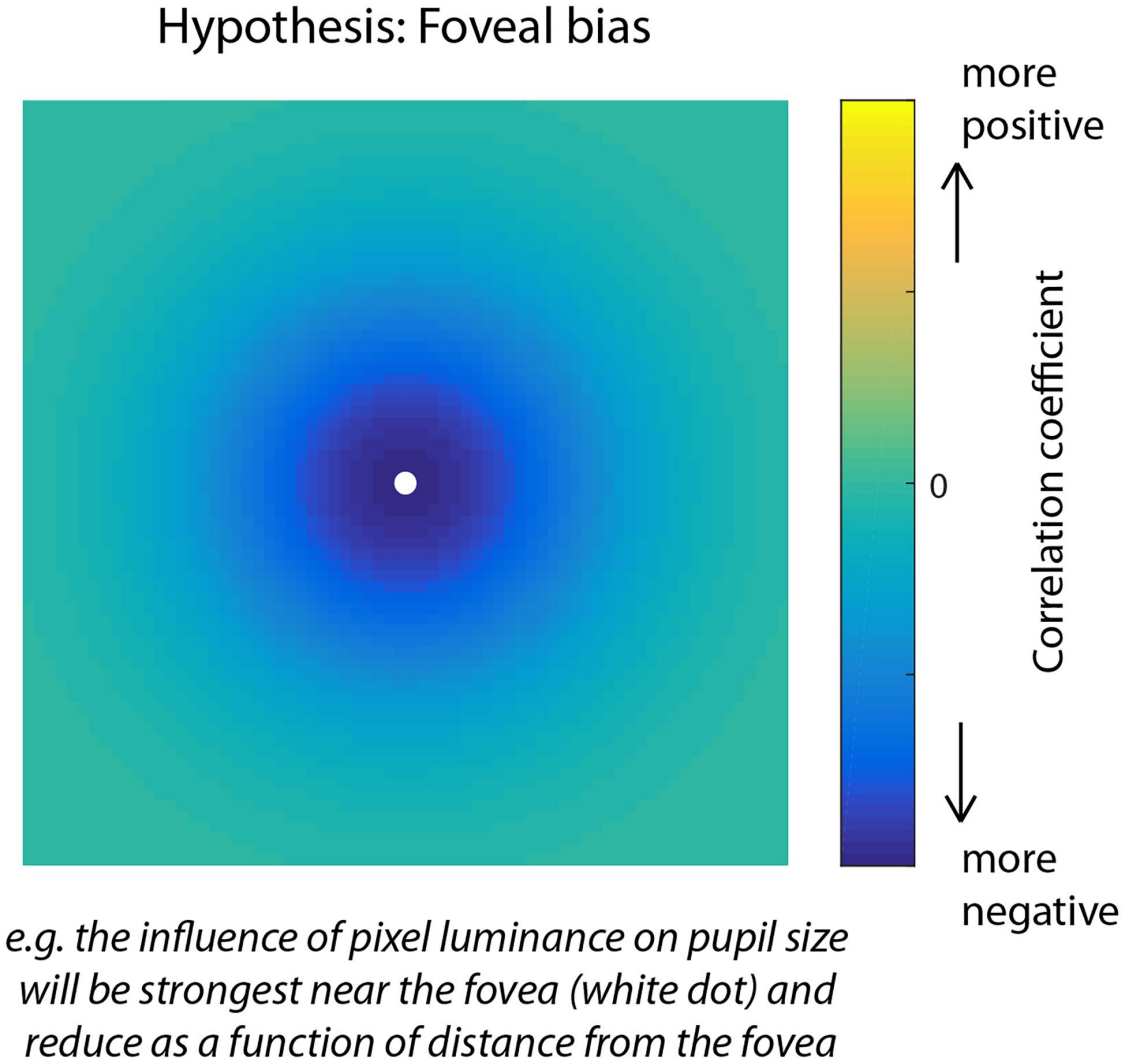
Visual representation of the foveal bias hypothesis for the correlation map analysis of Experiment 1. Due to the density of photoreceptors and sensitivity of the pupillary light response (PLR) to foveal vs. peripheral light stimulation, we predicted that pixels nearest fixation would correlate the most negatively with pupil size over time, and pixels further away would correlate less. The predicted negative correlation is due to the inverse relationship between light intensity and pupil size in which increases in luminance induce constrictions of the pupil.

Due to the inverse relationship between luminance and pupil size, where an increase in brightness causes a pupillary constriction and a decrease causes dilation, we expected a negative relationship between pupil size and RGB luminance and therefore that pixels with a stronger influence on pupil size would show larger negative correlations. Similar to other research areas, for example, that use reverse correlation to construct maps that reveal the influence of patterns of stimulus information on behavior (Gosselin and Schyns, 2001; Thurman et al., 2010; Thurman and Lu, 2013), we expected that the correlation map technique used here would be powerful enough to uncover fine-scale spatial patterns to characterize the influence of individual pixel intensities on pupil size. Consistent with research showing an effect of eccentricity such that the strength of the PLR is strongest for stimuli presented near the fovea and is systematically weaker for stimuli presented farther away, we hypothesized a foveal bias in the correlation maps (Figure 2) indicating that the visual system would pool luminance information from a focal region surrounding gaze position to modulate pupil size. An alternative possibility, however, would be that pixels were weighted in an anisometric pattern reflecting a broader contextual influence of luminance information on pupil size. In the absence of a specific theoretical prediction, an unexpected pattern, such as this would be interesting and informative, but would require *post hoc* analysis for interpretation.

### Results

#### Behavior

The analyses presented in this paper were focused on characterizing the relationship between pupil size and RGB scene statistics over the course of a visual search task in a virtual environment. Though our analyses are agnostic to behavioral performance in this task *per se*, it is relevant to report whether subjects were on-task and successful in reporting the correct number of target objects to be identified, and the correct answers to the mental math questions (see Task and Procedure). In terms of identifying and recollecting the number of targets (there were 15 target objects total for each condition), 25% of subjects reported exactly 15 targets but 71.9% of subjects did report at least 14 targets (range 5–32 targets). The variance could have been due to a misunderstanding by some subjects regarding which objects seen were supposed to be part of the target class they were assigned (Humvees, Motorcycles, Aircraft, or Furniture). On average, subjects had a mean accuracy of 75.2% in reporting the correct sum for the mental math questions and 94.3% of subjects got at least one of the three math questions correct. The behavioral results indicate that a majority of subjects were on-task in performing the visual search and mental math tasks as instructed.

#### Correlation Maps

**Figure 3:**
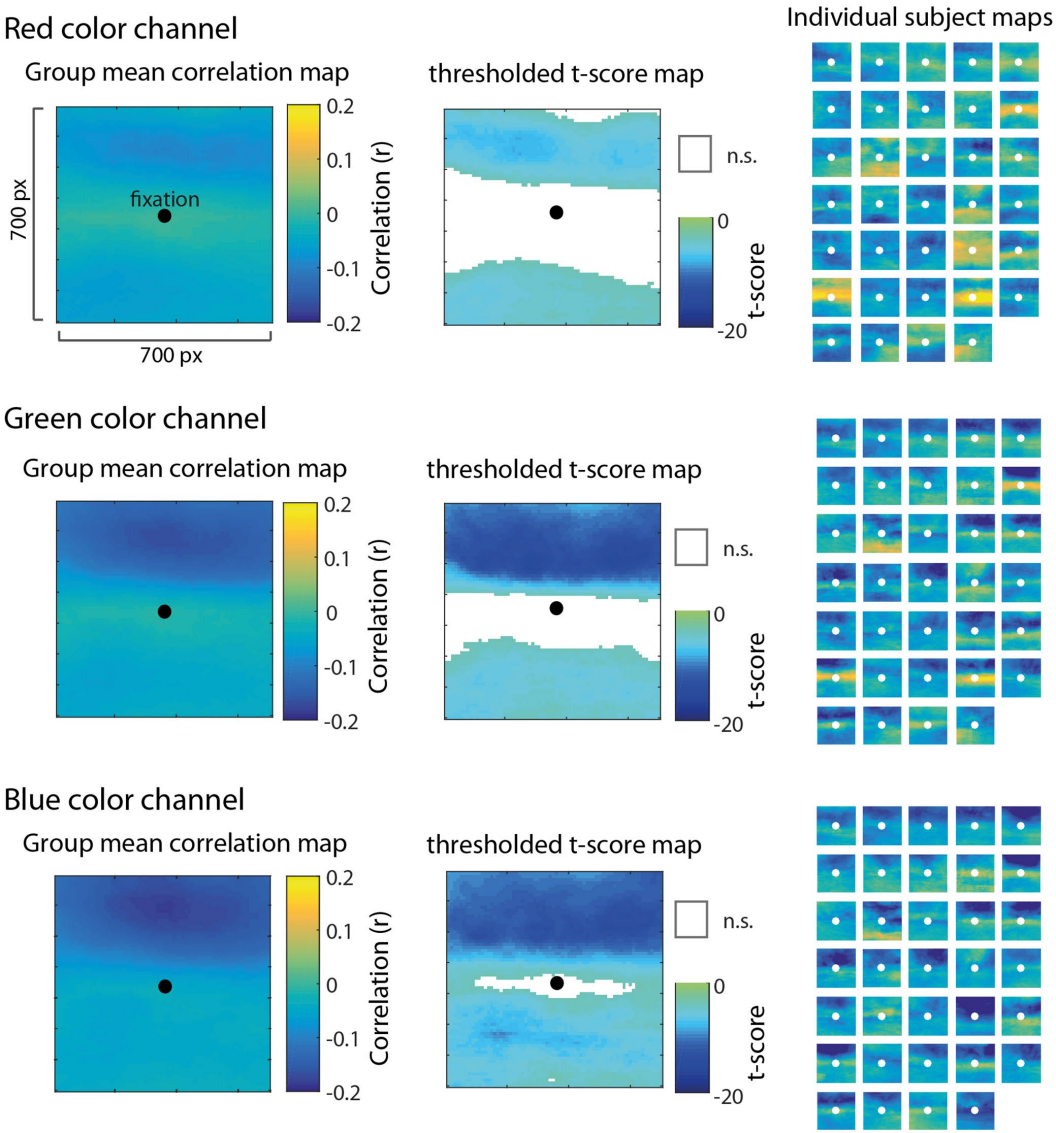
Results of the correlation map analysis for the red (top row), green (middle row), and blue (bottom row) channels, illustrating the correlation between fluctuations in pupil size and pixel intensity in a 700 × 700 pixel square region surrounding fixation. Group mean correlation maps (left) are shown with a black circle as a reference to indicate the center of gaze. We computed the *t*-score on Fisher Z-transformed correlation values for each pixel to derive statistical maps (center) and applied a false discovery rate threshold (FDR = 0.01) with non-significant pixels (n.s.) represented as white. Individual maps for each of 34 subjects (right) are shown to visually inspect the consistency of results across subjects.

The correlation map analysis allowed us to explore how different regions (at the pixel level) surrounding gaze position contributed to pupil size fluctuations relative to other regions. Critically, it does not assume a particular spatial pattern underlying the relationship between color luminance and pupil size; rather, the correlation maps allow us to discover patterns in the data. Figure 3 shows mean group-level correlation maps (left) for each color channel as well as thresholded statistical maps (*t*-scores; middle) highlighting pixels in which the distribution of correlation values between-subjects was significantly different from zero (FDR < 0.01). The subpanels in Figure 3 (right) show correlation maps derived from each individual subject to help visualize consistency of results between-subjects. We observed that the spatial patterns of these individual correlation maps were not as consistent from subject to subject for the red channel (Figure 3, top row), but were much more consistent for the green and blue channels (Figure 3, middle and bottom rows). In particular, the green and blue channels revealed a systematic bias showing a much stronger negative correlation for pixels above fixation compared to pixels below fixation. To evaluate this apparent upper bias in the blue channel for each subject statistically, we performed a *t*-test comparing the distribution of correlations above fixation to those below and found that 33/34 subjects showed a statistically significant difference (*p* < 0.05). There was no such consistency in the red channel across subjects, indicating a blue-green specificity for this upper visual field bias.

Contrary to the foveal bias hypothesis that predicted pixels nearest to fixation would correlate the most with pupil size (Figure 2), the correlation maps did not reveal an isometric influence of pixels in a circular region around fixation, nor did it show a pattern consistent with a non-linear weighting of pixels as a function of the distance from fixation (e.g., a Gaussian blob). Instead, the correlation maps showed the opposite pattern in which pixels closer to fixation were relatively less correlated with pupil size as evidenced by the statistically non-significant (n.s.) regions nearest to fixation in the thresholded *t*-score maps (Figure 3, middle column). Inspecting the individual subject maps, this result appears to be driven by inconsistency between-subjects in the central region, in which some subjects actually showed a positive relationship between pixel luminance and pupil size (yellow areas of the individual maps) and other subjects showed the opposite effect or, in most cases, a weak relationship in the central region. These results provide evidence decidedly against the foveal bias hypothesis and strongly support that contextual information outside the fovea can significantly modulate pupil size.

**Figure 4:**
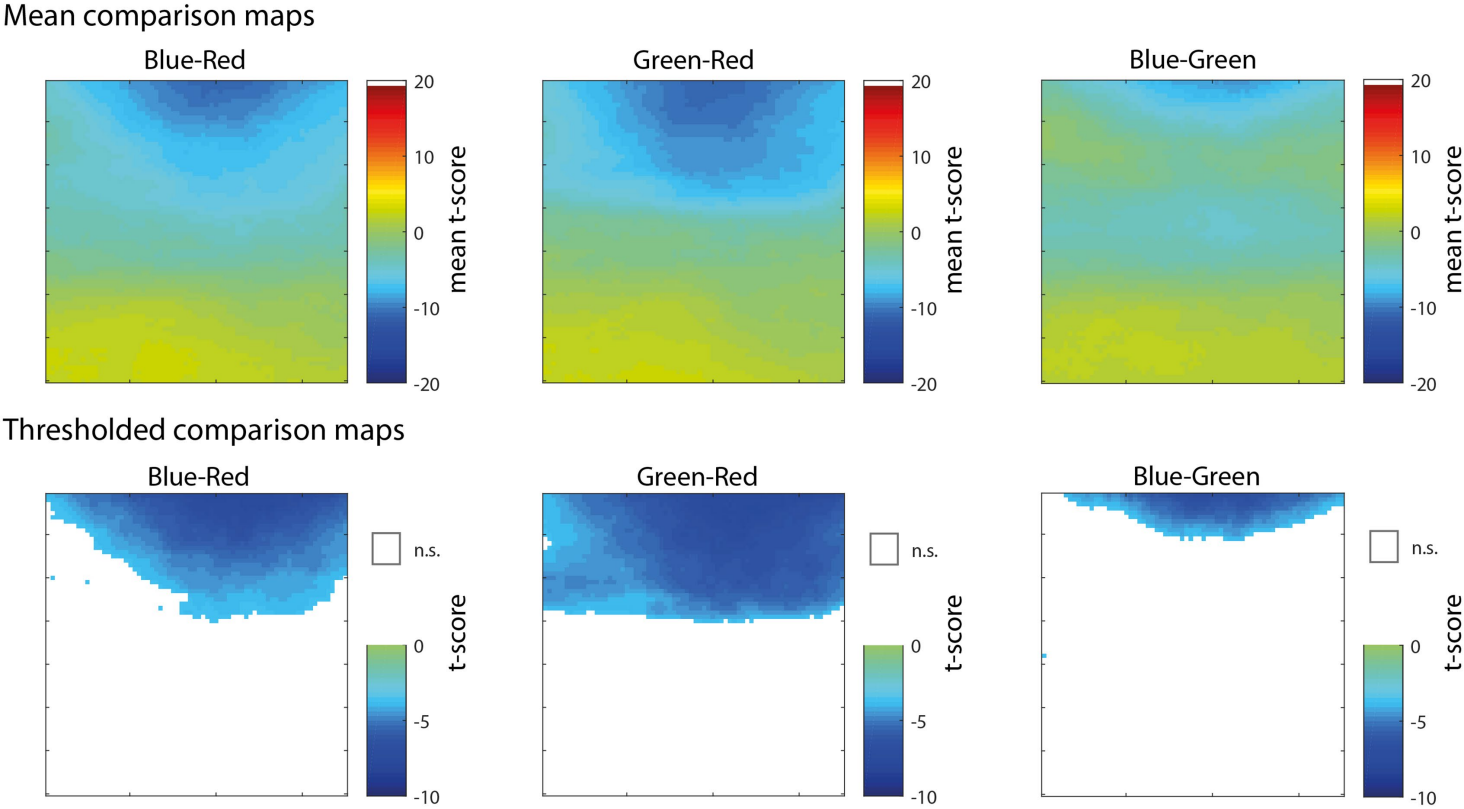
Maps showing pixel-wise *t*-scores (top row) for the comparison of correlations for dependent samples, comparing blue-red channel (left), green-red channel (middle), and blue-green (right). Thresholded versions of the same maps (bottom row) showing significant pixels (FDR < 0.01) colored by *t*-score according to the color map and non-significant pixels represented as white.

In the analyses presented above, the correlation maps illustrated pixels that were significantly correlated with pupil size with reference to the null hypothesis of no correlation (e.g., *r* = 0), but do not indicate whether the correlation for one color channel was significantly greater than another color channel. To compare between pairs of color channels, we performed a comparison of correlations for dependent samples that takes into account the within-subjects correlation of two variables (i.e., color channels) with a third variable (i.e., pupil size) as well as the correlation between the two variables (Williams, 1959; Steiger, 1980). As shown in Figure 4, the comparison of Blue-Red (left) and Green-Red (middle) revealed that the correlation of pixels in the upper part of the visual field was significantly more negative for both Blue and Green in comparison to Red. The comparison of Blue-Green (right) further showed that blue was significantly more correlated with pupil size than green for a smaller subset of pixels at the very top of the map. There were no significant differences among the color channels for pixels in the lower part of the visual field. This result further demonstrates that information in the blue and green channels, specifically located above fixation, had a dominant and disproportionate influence on pupil size that was much stronger than the red channel throughout the active visual search task.

Green and blue showed a similar pattern of results in the correlation maps, in part, because they were strongly correlated over time, specifically for pixels located in the sky region. In an attempt to isolate the influence of green vs. blue, we performed a follow-on analysis that converted the images from 8-bit RGB image space to CIELAB space, which is an alternative representation of an image in a three dimensional space that is more aligned with human perception. The three channels of CIELAB space include L* which is a representation of luminance on a scale of 0 (lowest luminance) to 100 (highest luminance), a* which is a representation of red-green chromaticity on a scale of −100 (more red) to 100 (more green), and b* which is a representation of blue-yellow chromaticity on a scale of −100 (more blue) to 100 (more yellow). We computed correlation maps using the same procedure as before, except in this case, we used pixel values represented in the three channels of L*a*b* space.

**Figure 5:**
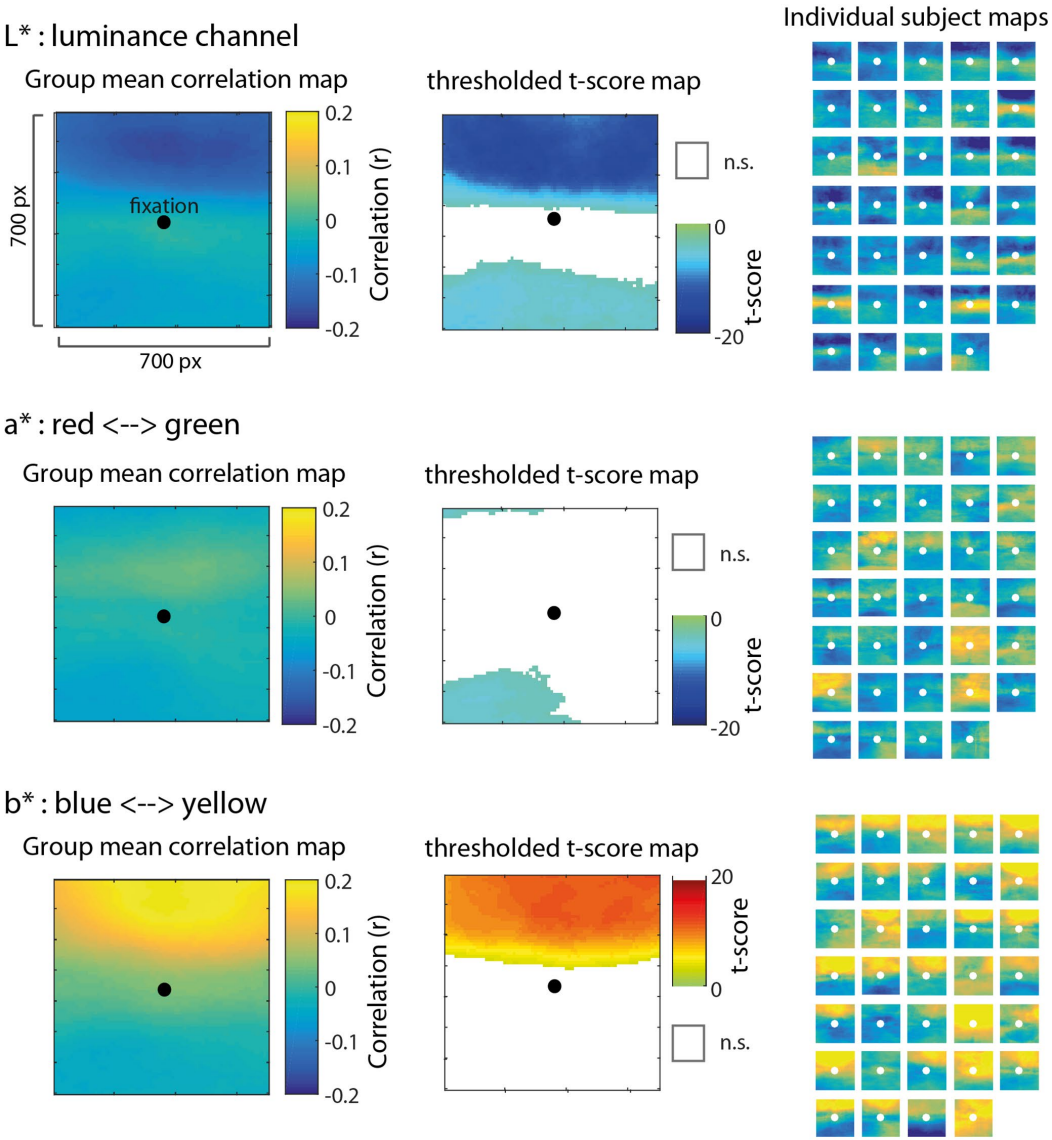
Results of the correlation map analysis for images transformed to CIELAB space with L* (top row), a* (middle row), and b* (bottom row) channels, illustrating the correlation with pupil size of each of the three dimensions in a 700 × 700 pixel square region surrounding fixation (similar to Figure 3). In CIELAB space, L* represents luminance on a scale of 0–100, a* represents chromaticity along the red (−100) and green (+100) axis, and b* represents chromaticity along the blue (−100) and yellow (+100) axis. Group mean correlation maps (left) are shown with a black circle as a reference to indicate the center of gaze. We computed the *t*-score on Fisher Z-transformed correlation values for each pixel to derive statistical maps (center) and applied a false discovery rate threshold (FDR = 0.01) with non-significant pixels (n.s.) represented as white. The positive correlation for pixels above fixation in the b* channel (bottom) indicates that decreases in pupil size (e.g., light-induced pupillary constrictions) were strongly associated with more negative values in b* space (e.g., increases in blue chromaticity). Individual maps for each of 34 subjects (right) are shown to visually inspect the consistency of results across subjects.

Results of this analysis are shown in Figure 5. The correlation map associated with the luminance channel (top row) showed a similar pattern to green and blue from the original analysis, in which the luminance of pixels above fixation had a statistically significant and disproportionate influence on pupil size. Figure 5 (middle row) shows that information in the a* channel was not very strongly correlated with pupil size, indicating that chromaticity along the red-green dimension was not a predominant signal influencing pupil size. By contrast, Figure 5 (bottom row) shows that information in the b* channel, representing chromaticity along the blue-yellow dimension, had a striking association with pupil size, particularly for pixels above fixation. The significant positive correlations in this map indicate that pupillary constrictions (reductions in pupil size) were associated strongly with increases in blue chromaticity (more negative values in b* space). This result provides additional clarity to the RGB results, and further evidence to suggest that there is a highly specific sensitivity of the PLR to visual patterns that indicate a blue sky is overhead.

## Experiment 2

In Experiment 1, we found that there was a stronger negative correlation between pupil size and blue pixels located in the top portion of images relative to fixation. We hypothesized this result as being associated with a “blue sky effect,” or a blue light from above effect (Suzuki et al., 2019). This hypothesis is motivated by three features of the visual system; first, that it is biased toward perceiving blue light as brighter; second, that it incorporates expectations (i.e., priors) of the structure of the environment such that visual patterns associated with sunlight from above are associated with an expectation of increased brightness; third, that the PLR has adapted to have increased sensitivity (indexed by a stronger PLR) to such patterns associated with sunlight from above (e.g., a blue sky).

To test whether the aforementioned effect can be explained by increased sensitivity to blue light above fixation, we designed a follow-up experiment that presented luminance-matched color stimuli (red, green, and blue) separated by either the horizontal or vertical meridian of the computer screen and paired with luminance-matched gray on the other side of the screen. We hypothesized that (i) blue would result in a larger constriction of pupil size due to a general bias or sensitivity to blue light, (ii) that blue light above would induce larger pupillary constrictions compared to blue light below, and (iii) that this effect would be specific for the blue channel and this orientation. These hypotheses are consistent with data presented in Experiment 1, and an ecological perspective that years of experience in the world with a blue sky (correlated with sun brightness) has adapted the system to anticipate or exaggerate the PLR specifically when blue is overhead.

### Participants

Thirty subjects with reported normal vision participated in this study. Due to the fact that interpolation introduces significant distortions in the shape of the PLR, we excluded trials in which a blink occurred in the first 1,500 ms following stimulus onset. Accordingly, we removed subjects from analysis if they had too many trials discarded due to ill-timed blinks according to the following criterium: (1) they must have had at least one valid (non-blink) trial for each condition (12 total conditions) and (2) at least 50% of trials overall must have been valid (non-blink). In total, 15 subjects (five females, 10 males, mean = 20.3 + 3.46 years) met these criteria and were included in the analysis. All subjects signed an informed consent form approved by the Institutional Review Board (ARL 20–014) prior to participation and completed a demographics questionnaire. The experimental protocol and human subjects procedures were in compliance with the Declaration of Helsinki.

### Task and Procedure

This data set was collected as a part of a larger data collection effort, where subjects first completed an attentional cueing task prior to completing the light from above task. The study had a total duration of 40 min and the task (described below) had a duration of 7 min. Only data from this specific task is reported here. After realizing that several early subjects were blinking too often during the first 1,500 ms of each trial, we modified our instructions asking subjects explicitly to withhold blinks for the first several seconds following stimulus onset. This resulted in less blinks during the critical period of the PLR for subsequent subjects.

**Figure 6:**
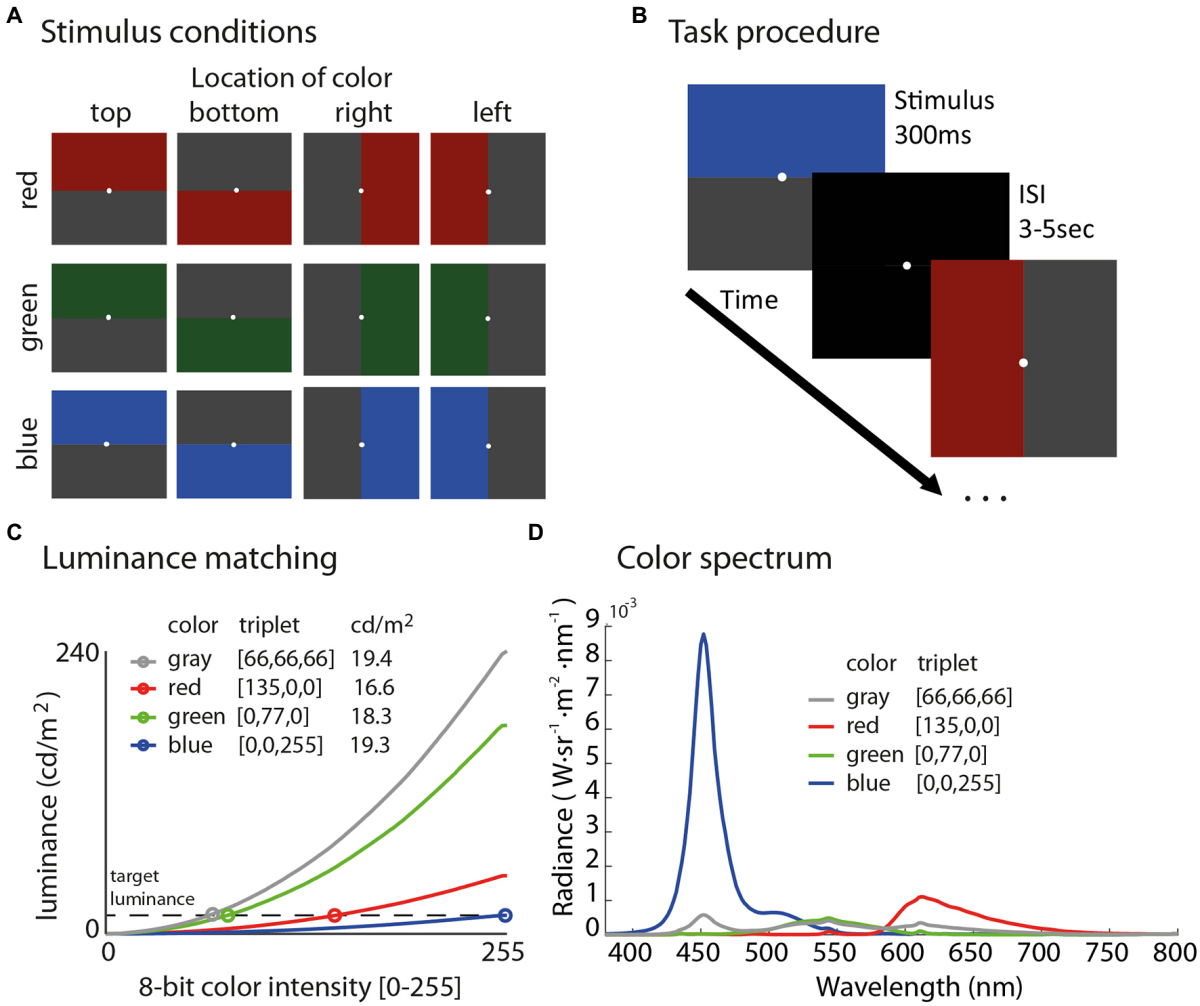
Illustration of all 12 stimuli in the experiment **(A)** sorted by color in rows and location in columns. Schematic of the task procedure **(B)** shows that subjects fixated on a white dot and were presented each stimulus for 300 ms followed by an inter-stimulus interval (ISI) with a black screen for 3–5 s. Stimuli were luminance matched using measurements from a spectroradiometer **(C)** to evaluate the specific influence of color and location on the PLR, while controlling for overall luminance between conditions, which ranged from 16.6 to 19.4 cd/m^2^. The color spectrum plot **(D)** shows radiance as a function of wavelength derived from the spectroradiometer for each color stimulus.

In this task, we split the computer screen along either the horizontal or vertical meridian, with one half of the screen colored gray and the other size colored with luminance-matched red, green, or blue. The order of stimulus presentation was pseudorandomized and counterbalanced by three color conditions (red, blue, and green), and four location conditions (top, bottom, left, or right) resulting in a total of 12 conditions (Figure 6A). A white fixation circle of 0.1 degrees was always present on the screen to help subjects maintain fixation. Each condition was repeated five times for a total of 60 stimulus presentations. Stimuli were presented for 300 ms followed by a black screen presented during the inter-stimulus-intervals (ISI) with a randomly jittered ISI between 3,000 and 5,000 ms (Figure 6B). The luminance of the stimuli was measured using the same spectrophotometer (SpectraScan Spectroradiometer PR-745) and protocol as in Experiment 1.

As shown in Figure 6C, all colors fell within the range of 16.6–19.4 cd/m^2^ [red (135,0,0) = 16.59 cd/m^2^, green (0,77,0) = 18.23 cd/m^2^, blue (0,0,255) = 19.28 cd/m^2^, and gray (66,66,66) = 19.43 cd/m^2^]. We chose this range of luminance on the basis of maximizing luminance in the blue channel to evoke the strongest possible PLRs; then, finding the RGB triplet in each color channel that was the best match to the maximum luminance of blue (19.28 cd/m^2^) based on our measurements with the spectroradiometer. Figure 6D shows the color spectrum plot for each stimulus used in the experiment.

The task was programmed in Matlab (2014a, The MathWorks, Natick, MA) and the Psychophysics Toolbox (3.0.14) was used for stimulus presentation (Brainard, 1997; Pelli, 1997). Stimuli were presented on a 2,560 × 1,440 Acer XB271HU monitor with a 120 Hz refresh (Windows 7 64-bit, Nvidia GeForce GTX 660 video card, Intel i7-4770K 4-core 3.5 GHz CPU, 16 GB RAM).

### Eye-Tracking Data Collection

Eye-tracking data were acquired in pupil-corneal reflection tracking mode (centroid pupil tracking) from the left eye, sampled at 1,000 Hz using the EyeLink 1,000 Plus eye tracker (SR Research, Ontario, Canada). Subjects were seated 75 cm from the monitor, while positioned in a chin rest to minimize head movements, and underwent a nine-point calibration procedure. Subjects recalibrated until an average validity error less than 1 deg. was obtained.

### Data Preprocessing

Pupil size data output from the EyeLink device is recorded in arbitrary units, so we first normalized pupil size on each trial to percent signal change by subtracting the pre-stimulus baseline, and then dividing by the baseline and multiplying by 100. The baseline was defined as the mean pupil size in the interval of 500 ms prior to stimulus onsets. We then averaged all valid trials (trials without blinks) associated with each condition within-subjects for subsequent group-level analyses.

### Data Analysis

Our analysis focused on characterizing and comparing the strength of the PLR, which is a ballistic constriction of the pupil initiated by the onset of a relatively brighter stimulus. Stimuli on each trial were always paired such that a colored stimulus (red, green, or blue) was presented with a luminance-matched gray (Figure 6A), so mean luminance across the entire screen (and between color conditions) was near constant (ranged from 16.6 to 19.4 cd/m^2^). Therefore, any difference in the strength of the PLR would be the result of differential sensitivities related to color spectrum, visual field location (top, bottom, left, and right), and/or an interaction between these two factors.

We quantified the strength of the PLR by identifying the minimum value (PLRmin = peak pupillary constriction) in the time interval from 0 to 2 s post-stimulus onset. We ran a repeated-measures ANOVA on PLRmin values with two factors including color (red, green, and blue) and color location (top, bottom, left, and right) to evaluate main effects and interaction effects between these factors. We corrected any violations from the assumption of sphericity with the Greenhouse-Geisser correction. Planned comparisons were also performed to assess the strength of the PLR specifically for top – bottom and left – right conditions to derive a set of difference scores for each color channel. We evaluated one-sample *t*-tests on these difference scores to assess whether the distribution was significantly different from the null hypothesis of zero. A difference score of zero would indicate that the relative location of the color did not impact the strength of the PLR, whereas a score significantly different from zero would indicate an effect due to the location of color (top compared to bottom or left compared to right). To correct for multiple comparisons (six total, two difference scores by three colors), we report adjusted *p*-values for *t*-tests using the Benjamini and Hochberg (1995) procedure to control the false discovery rate (FDR = 0.05).

### Results

**Figure 7:**
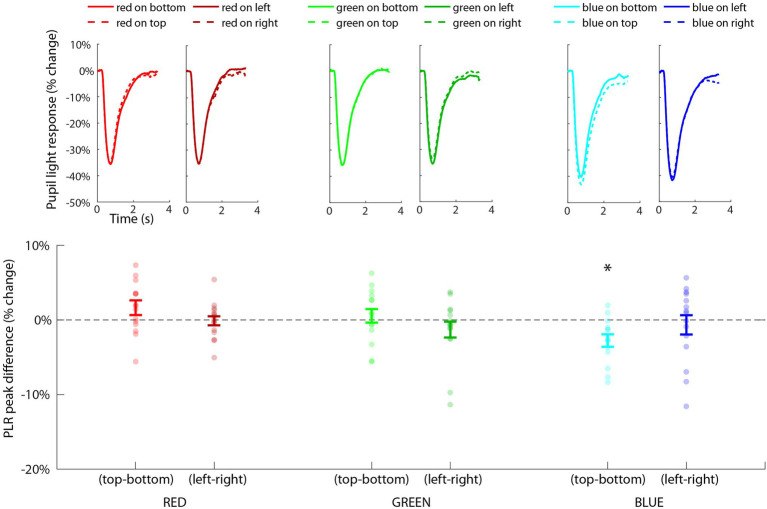
Results of Experiment 2 showing the PLR for each condition (top), organized by color and orientation (i.e., horizontal and vertical) along the x-axis. Statistical tests were conducted on the distribution of PLRmin difference scores (bottom) across subjects (i.e., individual dots) for each planned comparison of each type of color/orientation combination. The only significant effect (*p* < 0.02, FDR < 0.05) was the comparison of blue on top minus blue on bottom (indicated with *) showing that blue on top induced a significantly larger PLR than blue on bottom.

Pupillary light response waveforms for each condition are shown in Figure 7 (top), organized by color and orientation. The repeated-measures ANOVA revealed a significant main effect of color [*F*(2,28) = 43.67, *p* < 0.001, *η*^2^ = 0.56] due to blue being associated with a much stronger PLR (mean = −44.3%, SE = 2.0%) by comparison to red (mean = −37.7%, SE = 2.0%) and green (mean = −37.2%, SE = 2.0%). The main effect of location was not significant [*F*(3,43) = 0.76, *p* = 0.52], but we did find a significant interaction effect between color and location [*F*(6,84) = 2.72, *p* = 0.018, *η*^2^ = 0.03] indicating that the influence of color was also modulated by its location on the PLR.

To further probe this interaction effect, we conducted one-sample *t*-tests on the PLRmin difference scores for each color channel and orientation type (vertical or horizontal; Figure 7, bottom). Blue-top – blue-bottom was the only comparison that was significantly different from zero [*t*(14) = −3.60, *p* = 0.003, FDR < 0.05] indicating that the PLR was significantly greater specifically when blue was located on top compared to when blue was located on bottom. All other comparisons (red/top-red/bottom, red/left-red/right, green/top-green/bottom, green/left-green/right, and blue/left-blue/right) were non-significant (all values of *p* > 0.05, FDR > 0.05). This effect could not be explained by baseline differences in pupil size prior to the stimulus, as the 500 ms mean pre-stimulus baseline pupil size for blue-top was 913 a.u. (*SD* = 256 a.u.) and for blue-bottom was 926 a.u. (*SD* = 282 a.u.), and the *t*-test indicated a non-significant difference in baseline pupil size, *t*(14) = 0.53, *p* = 0.6. In fact, there was a non-significant difference in baseline pupil size for all comparison pairs (*p* > 0.05), ensuring that differences in baseline pupil size could not explain this pattern of results. This highly specific effect for blue was consistent with our hypotheses that the response of the pupil to blue light would be greater than red and green, and also that blue on top would induce a significantly larger PLR than blue on bottom. The specificity of this result is consistent with the hypothesized blue sky effect and provides a potential explanation for the significant contextual effect in Experiment 1, in which blue pixels above fixation had the strongest modulatory effect on pupil size.

## Discussion

In this study, we leveraged a unique data set in which subjects performed a navigation and active visual search task in a complex 3D virtual environment to examine the relationship between fluctuations in pupil size and dynamic visual scene statistics. Our primary goal was to better characterize how the pupil is influenced by rapid changes in complex luminance patterns associated with realistic scenes. We did this by correlating pupil size and pixel intensities in the RGB channels of the image in relation to the center of gaze as subjects actively performed the task. A primary motivation for this work is that while there are many published studies that have characterized the PLR in controlled laboratory experiments, most of these studies use simplistic stimuli and longer stimulus durations that do not match the complexity and high temporal rate of change of luminance in realistic visual scenes. It is unknown the extent to which findings from these studies will generalize to complex environments to enable effective use of cognitive pupillometry in less constrained task environments and real-world applications. We tested whether three findings from previous literature on the relationship between pupil size and luminance would generalize to a naturalistic task: (i) pupil size is more strongly influenced by luminance inputs near the fovea, (ii) pupillary responses to light are modulated by color spectrum effects (given equal luminance), with a specific sensitivity to blue light, and in Experiment 2, and (iii) that pupil size is more strongly influenced by blue light, specifically when it is located above fixation.

### No Fovea Bias

We hypothesized a foveal bias in the correlation maps, consistent with research showing that stimuli nearer to fovea induce stronger PLRs than stimuli in the periphery (Crawford and Parsons, 1936; Legras et al., 2018; Hu et al., 2020). This prediction is also related to the structure of the human visual system as the distribution of photoreceptors changes dramatically as a function of distance from the fovea, and there are significant differences in visual function between foveal, parafoveal, and the peripheral regions (Preuschoff et al., 2011; Strasburger et al., 2011). Previous research has shown that cone mediated pupil responses to photopic light (i.e., simple circular patches of light stimulus) are maximal up to 7 degrees of visual angle, and has reasoned that the decrease in response may be due to the drop off in cone density after 7 degrees (Legras et al., 2018; Kelbsch et al., 2019). Our data, however, did not support this hypothesis, instead revealing a robust and unpredicted pattern in the correlation maps. Pixels very close to gaze position actually tended to correlate the least strongly with pupil size. This pattern was most prominent for the red channel, in which all of the pixels within about 2–3 degrees from fixation were not significantly associated with pupil size, but it was also apparent in the blue and green channels.

### Blue Sky Effect

The correlation maps instead showed evidence to support a strong contextual influence on pupil size associated with the presence of blue occurring above fixation in the environment, that we refer to as the “blue sky effect.” Our finding that blue varies more strongly with pupil size compared with red or green (Figure 4) is consistent with previous research. For example, the Helmholtz-Kohlrausch effect indicates that color saturated light is perceived as being brighter than light that is less saturated at equiluminance, and that this effect is increased for particular wavelengths including blue (Wood, 2012; Suzuki et al., 2019). Likewise, eye-tracking studies find that a perceived increase in brightness coincides with pupillary changes (Sulutvedt et al., 2021), with a stronger effect for blue light (Suzuki et al., 2019).

In addition to the finding that blue light enhances the PLR, we found that this effect was specific to occurring above fixation. This result was highly consistent across subjects as observed in the individual correlation maps for the green and blue channels (Figure 3, right). While location of fixation was not experimentally manipulated or controlled in this analysis, these results indicate that fixations toward the horizon may have comprised the majority of fixations. When this well-matched visual pattern of blue on top and terrain on bottom fell onto the retina, it induced robust pupillary responses that accounted for a large degree of variance in the pupil signal by comparison to other areas or features of the environment. One surprising aspect to this finding is that the blue sky itself had lower luminance intensity compared to the terrain below, which was composed of more red and green hues that tend to carry stronger luminance signals (See Supplementary File 1). So while the strength of the luminance signal tended to be stronger below fixation, it was the specific temporal pattern of blue luminance above fixation that correlated most with pupil size, indicating the strength of this contextual effect.

### Confirming the “Blue Sky Effect”

To confirm this finding and evaluate whether the “blue sky effect” has a more generalized effect on the PLR, we ran a second experiment with a controlled task to assess whether the PLR is indeed stronger, specifically, when blue is seen above fixation. The experimental procedure was careful to match the luminance level across color stimuli (gray, blue, red, and green). The only variables that were manipulated on each trial were the color presented (paired with gray) and its location (on top, bottom, left, or right). Results showed that the PLR was indeed stronger for blue stimuli in comparison to green and red, which replicates results from prior studies (Suzuki et al., 2019). Importantly, the results also showed a specific effect in which the PLR was strongest when blue was presented on top compared to on bottom. There were no other statistically significant effects associated with the relative location of other color stimuli. This result provides additional support to the blue sky hypothesis, suggesting that the PLR is influenced most strongly by blue light, and in particular, when blue light is present above fixation.

An evolutionary and ecological perspective can provide a speculative explanation of the observed blue sky effect on the PLR. A primary function of the pupil is to constrict in response to a sudden bright stimulus and to dilate in response to relatively dark stimuli in order to maintain optimal acuity under a wide range of visual conditions, and perhaps also to protect the photosensitive retina from very strong levels of brightness (Mathôt, 2018). Through lifetimes of experience on earth, a mechanism may have evolved within the circuit that controls the PLR to be particularly sensitive to visual patterns that indicate a person is outdoors in sunny, daytime conditions, and to constrict in anticipation of the subsequent strong levels of brightness (Laeng and Endestad, 2012; Zavagno et al., 2017; Suzuki et al., 2019). While the present study cannot shed light on the purpose or specific mechanisms supporting the observed heightened sensitivity of the pupillary system to blue light from above, future studies may be designed to better ascertain the underlying mechanisms driving this effect. For example, it would be informative to test whether other species show a similar sensitivity to blue light from above or to examine the strength of this effect in special populations, such as those with color blindness or other visual disorders. If instead this effect were merely due to unequal distributions of short wavelength sensitive photoreceptor cells in the retina, then we might expect between subject variability in receptor density to correlate with the strength of the blue light from above effect on the PLR.

### Practical Implications

There remain substantial challenges to enabling reliable inference of pupil-linked states in complex or uncontrolled visual environments outside the laboratory due to the fact that multiple neural systems combine to influence the unitary pupillary signal (the sequence of constrictions and dilations over time). Change in pupil size can reflect mental states over a range of temporal scales, from transient processes related to attention (Nieuwenhuis et al., 2011; Preuschoff et al., 2011; Geva et al., 2013; van den Brink et al., 2016) and decision making (Einhauser et al., 2010; Cavanagh et al., 2014; Hoffing et al., 2020) that unfold over a few seconds, to more general arousal and fatigue states (Franklin et al., 2013; Hopstaken et al., 2015; Unsworth and Robison, 2018) that unfold over a longer time period (Aston-Jones and Cohen, 2005). As a result, it is difficult to disentangle whether the pupillary signal at any given point in time reflects the influence of a cognitive or non-cognitive factor (Mathôt, 2018). One potential approach to tackle this challenge is to develop appropriate models to best account for complex luminance signals on pupil size and then examine the residual unaccounted for variance to better estimate cognitive-based effects.

The observed blue light from above effect on pupil size has practical implications for analysis of pupillary data in complex virtual environments and in real-world scenarios. For future, real-world systems that aim to measure environmental light to account for the influence of non-cognitive luminance effects on pupil size, it may be pertinent to ensure that blue light is captured and modeled appropriately relative to other wavelengths to ensure the best prediction of light-induced pupillary fluctuations. It could also be the case that a sensor specifically tuned to blue wavelength light may be sufficient for some applications. There is, however, still much work to fully understand the efficacy of subtractive models that attempt to better characterize cognitive influences on the pupil by subtracting or factoring out estimates of non-cognitive influences. We believe that a major contribution of this work is in rejecting the foveal bias hypothesis for estimating the influence of luminance on pupil size in complex visual environments. It does not appear to be the case that the visual system uses an area around the high density fovea as a sensor to pool luminance information for driving the PLR. The system instead seems more sensitive to global patterns of information and contextual cues, like the blue sky effect and perhaps others, in determining the appropriate pupil response to light in a given environment.

For controlled studies in cognitive pupillometry, this also suggests a prime importance for controlling the level (and visual patterns) associated with blue light in particular. Experimenters should be careful to counterbalance conditions if color stimuli are used with variation along the color spectrum to avoid confounds that might influence interpretation of phasic pupillary responses to cognitive events. Such color spectrum effects should also be accounted for (or anticipated in the analysis) in these studies as equating luminance alone may not have the desired effect of equating the strength of the pupillary light response to differently colored stimuli. As shown here, the pupil response to a blue stimulus is significantly larger than to a red stimulus with well-matched luminance. As our data show, even the relative location of the blue stimulus (above or below fixation) can impact the strength of the pupil response. Further care should be taken in interpreting pupillary data, especially when examining pupillary responses to naturalistic or more complex stimuli that match the spatial structure of the blue light from above effect (e.g., blue predominantly in the upper part of the image).

### Limitations

There are some limitations associated with the current study. The analyses in Experiment 1 focused on examining correlations (i.e., linear associations) between pupil size and RGB luminance over a rather long period of time (up to 15 min of data or 8,000–10,000 contiguous data points). While this approach is well-suited for capturing longer timescale relational trends in the data, this analysis technique does not have sufficient temporal resolution to capture how momentary changes in luminance and/or scene characteristics influence ballistic changes in pupil size at short timescales (e.g., on the order of seconds or milliseconds). Our correlational analysis does show that increases in RGB luminance tend to be associated with reductions in pupil size (and vice versa), as expected, but this approach does not shed light on the precise relationship between sudden changes in complex luminance patterns, whether due to eye movements or image dynamics, and the precise shape of the PLR. It is unclear the extent to which existing parametric models of the PLR to single transient changes in luminance, such as the gamma function (Korn and Bach, 2016), would extend to the rich, dynamic image sequences seen in a virtual environment like the one used in the current experiment. We did incorporate the expected time delay between luminance and pupil size (empirically derived to be about 500 ms) in our correlational analyses, but did not convolve dynamic luminance patterns with a specific parametric impulse response function. It is unclear whether such models would better account for short timescale pupillary fluctuations and impact results of the current study, but poses an interesting question for future research.

Because the task was performed in a virtual environment of our design, which can be described as a mountainous desert wasteland landscape, the set of images seen by subjects on the computer screen tended to have a particular spatial structure and spectral composition. The pattern of results we observed in Experiment 1 are clearly influenced by this structure. For example, the correlation maps show correlation values that vary primarily in the vertical direction, but not the horizontal direction, likely due to the structure of the images themselves. In a horizontal slice of the image near the bottom there will tend to be more red, brown, and grayish colors associated with the terrain and less variability from left to right. A horizontal slice in the upper part of many images would often consist predominantly of blue color associated with the sky, as long as subjects are looking toward the horizon (which was likely a common focal point for subjects as they navigated the virtual environment). If the experiment were run in a different virtual environment or even a real-world environment, it is likely that the structure of the correlation maps would change to reflect the prevailing spatial and spectral structure of those images. While this does not greatly impact the main finding in this paper that supported the contextual effect of the blue sky on pupil size, we would expect differences in the structure of correlation maps depending on properties of the visual environment if future studies use this same approach.

Related to the complexity of the task, there is no doubt that various cognitive processes related to visual search, navigation, covert attention, working memory, etc., were ongoing throughout the task that lasted several minutes. Because, we did not control these factors in the experimental design, as it was designed to be a free and active task, we had no way to account for such factors as covariates in the analysis – a situation that is pervasive in real-world cognitive pupillometry. The perspective of our correlation analysis was to essentially treat these cognitive processes as “noise” in the pupillary signal that would influence pupil size independently (and likely to a lesser extent) with respect to luminance information. We do not believe that cognitive effects could explain the robust negative association we found between pixel luminance and pupil size in this task, nor can they explain the reported blue sky effect in the correlation maps. If anything, the correlations we measured are likely an underestimate of the actual strength of the influence of luminance on pupil size because of the unaccounted for “noise” introduced by continuous cognitive processes.

A subset of our participants were older than 60 years (*n* = 4), and with age there is a tendency for yellowing of the eye’s lens and changes in the perception of color, particularly blue light (Gaillard et al., 2000; Michael and Bron, 2011). We re-ran our analyses only for subjects with ages less than 60 years (*n* = 30) and found that the pattern of results and level of statistical significance was not greatly impacted. In fact, all four of the subjects over 60 years showed a blue sky effect (significantly stronger negative correlation for blue pixels above fixation versus below). A recent study by Rukmini et al. (2017) showed that while the strength of the pupillary light response is reduced overall with aging, the amount of reduction was similar for red (631 nm) and blue (469 nm) wavelength light. They concluded that yellowing of the lens does not selectively reduce melanopsin-dependent light responses as reflected by the pupillary light response for people over 60 years old. Our data are consistent with this finding.

Lastly, in Experiment 2, we were not able to perfectly equate luminance across colored stimuli (Figure 6C). Based on the measurements, we obtained with the spectroradiometer in the lab, we were only able to match luminance in a range from 16.6 to 19.4 cd/m^2^, which is a very narrow range compared to the full spectrum (which ranges from 0–240 cd/m^2^), but the possibility remains that the response to blue stimuli was larger than red in Experiment 2 because of the luminance difference of 2.8 cd/m^2^. The dose-response curve is not well mapped out for PLR amplitude and incremental changes in luminance for different wavelengths of light, so we believe the possibility remains that the difference between red and blue could be due to luminance *per se*, and not a color specific phenomenon in Experiment 2. We will note that this potential confound does not affect our interpretation of the orientation-specific blue sky effect because we compared PLRs to stimuli within each color that were equiluminant and only varied by the location of the color.

## Conclusion

In this study, we examined correlations between pupil size and dynamic image statistics in the context of a free visual search and navigation task in a 3D virtual environment. We found that blue and green pixel intensities had a disproportionately large impact on pupil size in comparison with the red color channel. Furthermore, we found that visual scenes in which blue was predominantly overhead had the strongest influence on pupil size, which led us to hypothesize a “blue sky effect.” We conducted a follow-up controlled laboratory experiment and found evidence consistent with our hypothesis, showing a specific sensitivity of the PLR to blue light when it is located above fixation. From an ecological perspective, we speculate that the heightened sensitivity of the pupillary system to this visual pattern may be a useful adaptive response due to the persistent association between sunlight, large increases in brightness, and the blue sky in our daily lives. From a practical standpoint in terms of pupillometry research, the findings of this report suggest that equating luminance alone may be insufficient to account for luminance effects on pupil size if multi-colored stimuli and/or naturalistic images are used in psychological research. More research is necessary to fully understand how best to account for the influence of light on pupil size for studies or applications in complex visual environments outside the laboratory.

## Data Availability Statement

The raw data supporting the conclusions of this article will be made available upon request via email by the authors, without undue reservation.

## Ethics Statement

The studies involving human participants were reviewed and approved by ARL Institutional Review Board. The patients/participants provided their written informed consent to participate in this study.

## Author Contributions

ST conducted the analysis for both experiments. SG and JT designed Experiment 1. ST and RH designed Experiment 2 and wrote the first draft of the manuscript. AM and AR collected data for Experiment 2. AM, AR, JT, and SG contributed to manuscript revision. All authors contributed to the article and approved the submitted version.

## Funding

This research was sponsored by the Army Research Laboratory and was accomplished under Contract Number 1782 W911NF-10-D-0002.

## Conflict of Interest

SG is employed by DCS Corporation (United States).

The remaining authors declare that the research was conducted in the absence of any commercial or financial relationships that could be construed as a potential conflict of interest.

## Publisher’s Note

All claims expressed in this article are solely those of the authors and do not necessarily represent those of their affiliated organizations, or those of the publisher, the editors and the reviewers. Any product that may be evaluated in this article, or claim that may be made by its manufacturer, is not guaranteed or endorsed by the publisher.
