# Supplementary material for: “Blue Sky Effect”: Contextual Influences on Pupil Size During Naturalistic Visual Search
Source: Front Psychol. 2021 Dec 21;12:748539. doi: 10.3389/fpsyg.2021.748539 (PMC8725886; doi:10.3389/fpsyg.2021.748539)
Supplement: Supplementary file 1 [file Data_Sheet_1.PDF]

Supplementary File 1

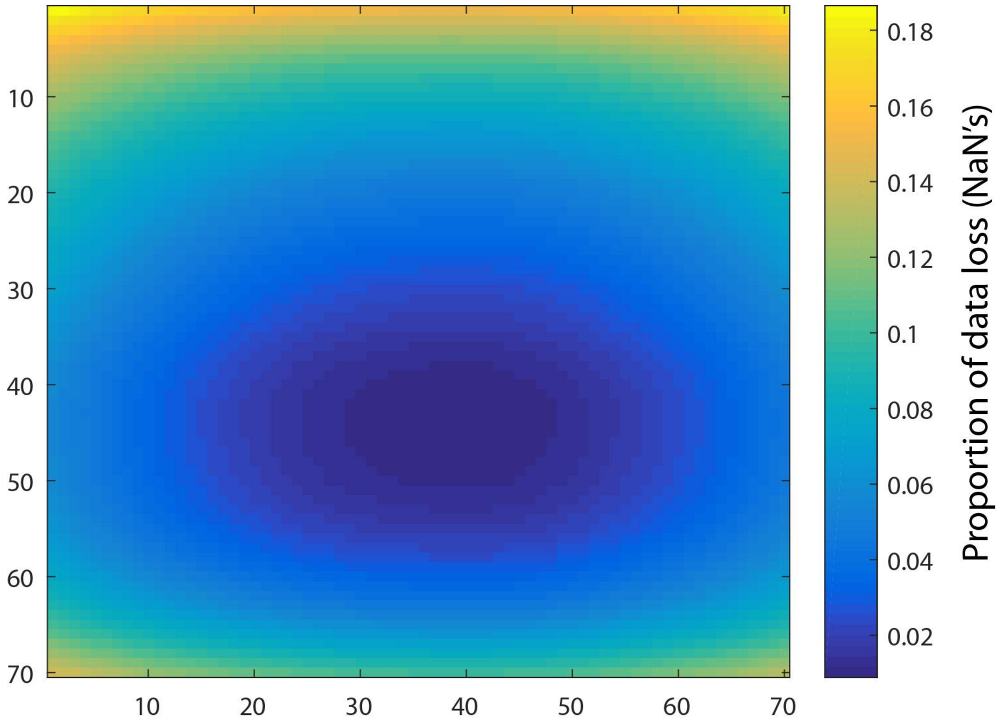

Supplementary Figure 1. Image map showing the proportion of data loss, represented as NaN's (not a number in Matlab), due to fixations near the edge of the screen. Since we analyzed a 700x700 pixel window (downsampled to 70x70 pixels) around gaze position at each time point, any fixation within 350 pixels of one of the screen edges resulted in a lack of visible image pixels to be represented and was therefore not included in the correlation analysis. The mean overall amount of data loss due to this issue was 6.47% and the peak amount of data loss occurred near the uppermost edge of the image space, representing 18.1% of the data. The range of data loss above fixation (defined as the center of this space) where the blue sky effect was most significant was approximately 6-12%. We do not believe that this pattern of data loss can explain the robust effects we observed in the correlation maps (shown in Figures 3-5).

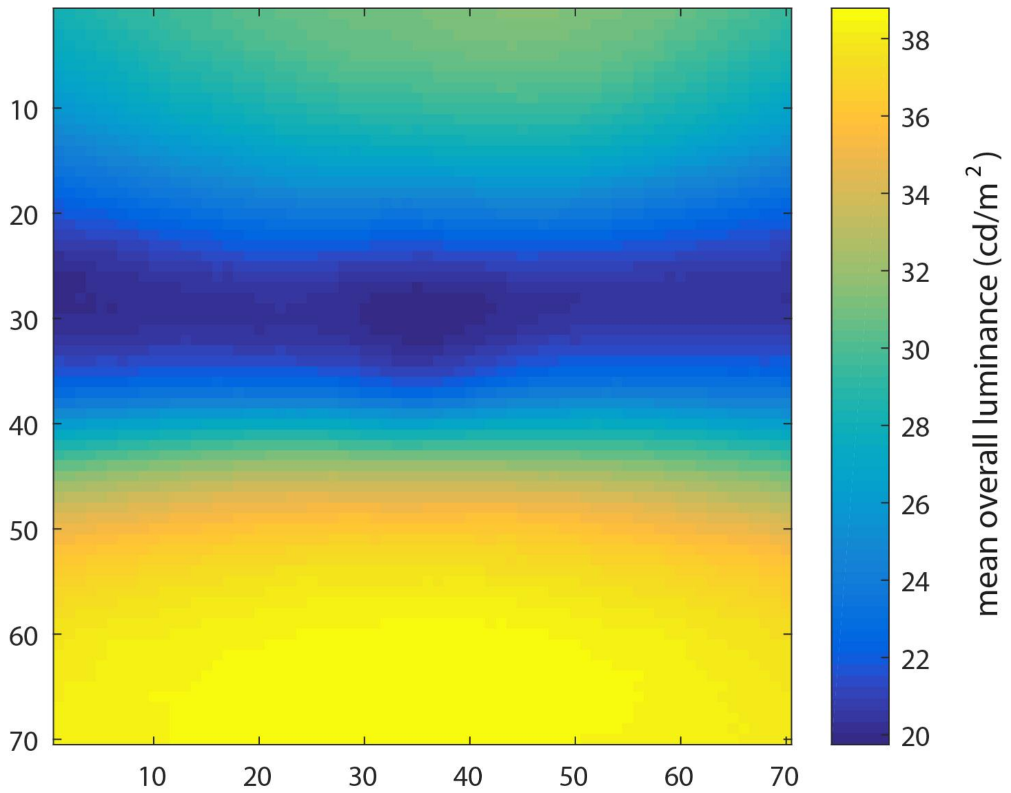

Supplementary Figure 2. Image map showing mean luminance over space derived from all 700x700 image windows (downsampled to 70x70 pixels) centered on gaze position across all subjects (n=34). The terrain, which was present predominantly below fixation (the center of this space), had significantly higher mean luminance than the sky often present above fixation. This pattern is in contrast to our correlation maps (seen in Figure 3), which showed that image pixels above fixation in the blue channel were the most strongly correlated with pupil size over time, despite not being the brightest region of the screen, on average. This discrepancy highlights the influence of the contextual blue sky effect on pupil size.

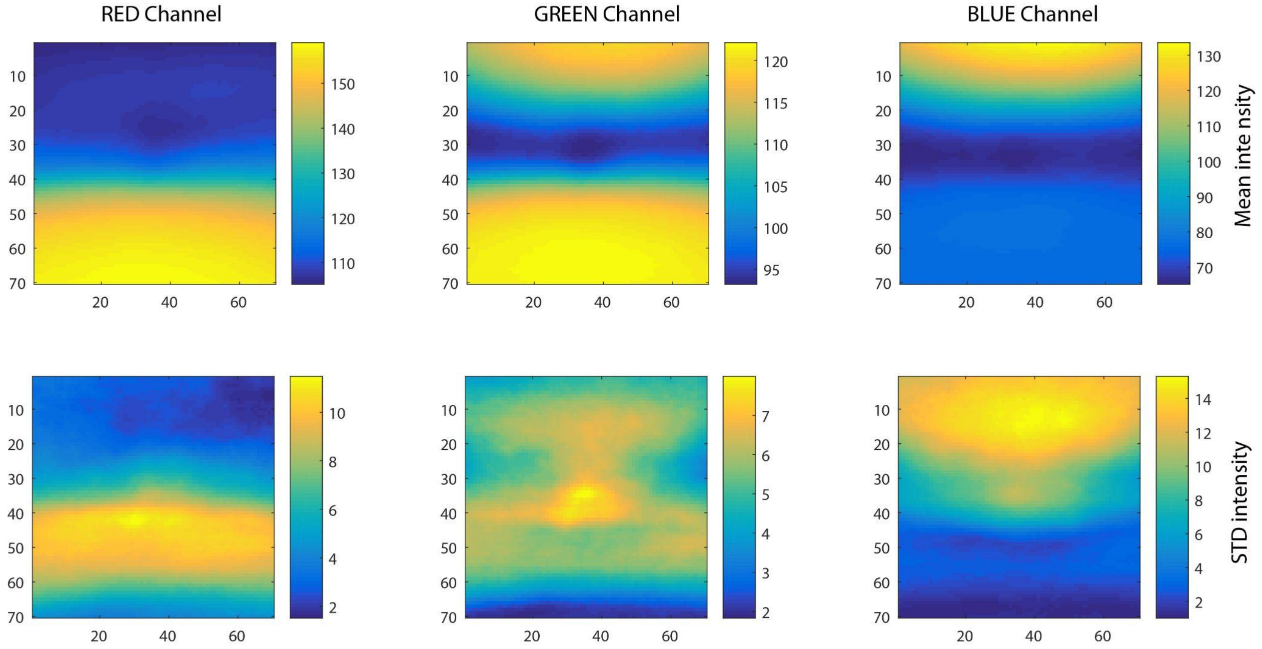

*Supplementary Figure 3. Image maps showing the mean 8-bit image intensity in the red, green and blue channels (**top**) across all frames and all subjects in a 700x700 window (downsampled to 70x70 pixels) around fixation. There was a relatively higher intensity in the red channel for pixels below fixation due to the predominance of red in the desert-like terrain and lack of red in the sky. There was a relatively higher intensity in the blue channel for pixels above fixation due to the frequent presence of the sky. Information in the green channel was present both in the sky and terrain. The horizontal band of relatively low intensity across all three color channels likely corresponds to characteristics of image scenery near the horizon, a common focal point in the visual search and navigation task. Images in the bottom row illustrate the standard deviation of 8-bit RGB intensity across all frames and subjects.*

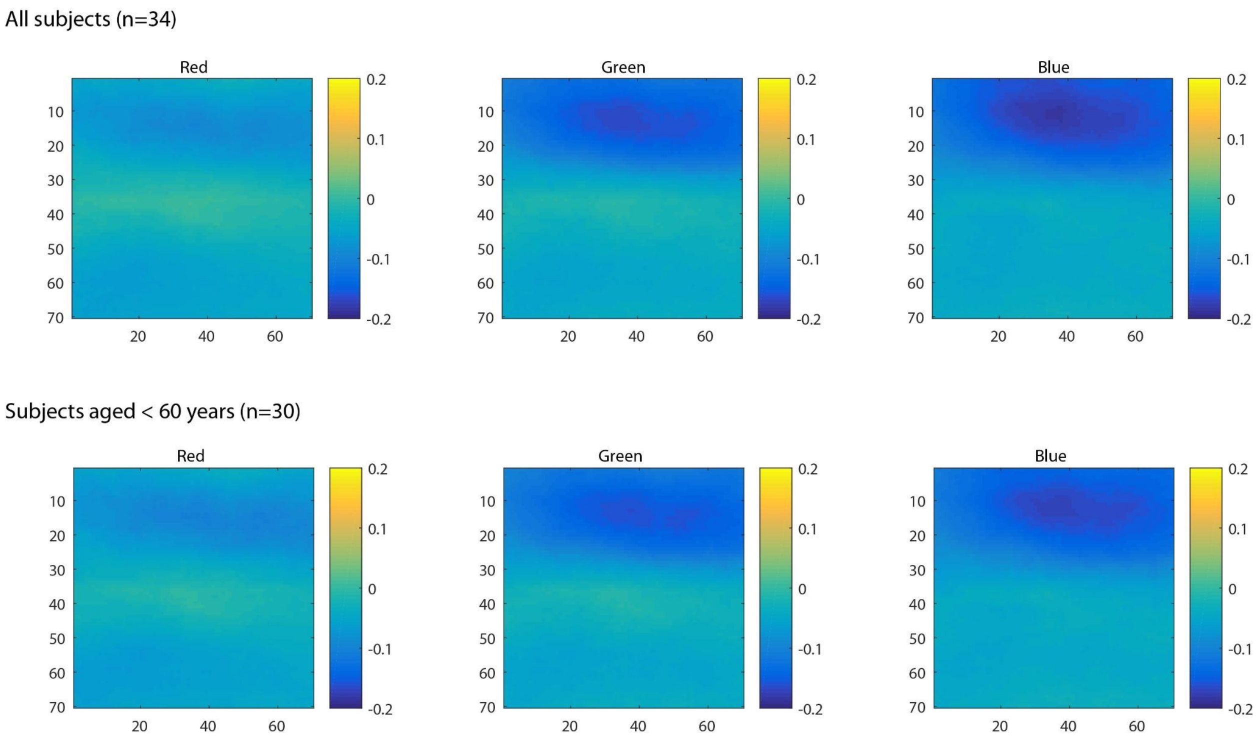

*Supplementary Figure 4. Correlation maps showing results of the correlation of each pixel with pupil size for all subjects (**top; as reported in Figure 3 of the manuscript**), and correlation maps derived from the subset of subjects with age less than 60 years (**bottom**). The pattern of results was not impacted by including or removing these four subjects from analysis, so we included all subjects in the final analysis reported in the manuscript.*
